# Observations on a leptospirosis case with ocular determinations

**DOI:** 10.1186/1471-2334-13-S1-P43

**Published:** 2013-12-16

**Authors:** Lucian Negruțiu

**Affiliations:** 1 Clinic of Infectious Diseases I, Timişoara, Romania

## Background

Leptospira infection or icterohemorrhagic disease (LIR) are not rare entities. Our study introduces a patient infected with LIR following repeated professional underwater dives.

## Case report

Because of disturbances in visual acuity, the patient initially turned to the services of the Timişoara Ophthalmological Clinic in 2011, and was later sent to the Infectious Diseases Clinic of Timişoara, showing low grade fever, moderate hepatosplenomegaly, discreet subicteric sclera and a severely increasing deterioration of visual acuity.

Direct ophthalmoscopy revealed disseminated bilateral chorioretinitis foci. The Infectious Diseases Clinic Laboratory confirmed hematological and serological diagnosis of LIR.

## Conclusion

The ocular chorioretinal determination of LIR is not frequent. In such situations, inoculation particularities along with evolving clinical and biological characteristics invite the clinician to extend the area of investigation.

